# Development and external validation of a mixed-reality aneurysm clipping simulator

**DOI:** 10.1007/s10143-025-03846-x

**Published:** 2025-10-09

**Authors:** Matthias Gmeiner, Andreas Schrempf, Thomas Thurner, Wolfgang Fenz, Bertram Sabrowsky-Hirsch, Michael Giretzlehner, Robert Prückl, Stefan Schaffelhofer, Zoltan Major, Sebastian Lämmermann, Melanie Baumgartner, Lukas Drabauer, Jozsef Nagy, Giuseppe Esposito, Elisa Colombo, Nico Stroh-Holly, Andreas Gruber

**Affiliations:** 1 Department of Neurosurgery, Kepler University Hospital, Johannes Kepler University Linz, Wagner-Jauregg-Weg 15, 4020 Linz, Austria; 2 Clinical Research Institute for Neuroscience, Johannes Kepler University Linz, 4020 Linz, Austria; 3 Research Group for Surgical Simulators Linz, Upper Austria University of Applied Sciences, 4020 Linz, Austria; 4 RISC Software GmbH, Unit Medical Informatics, Softwarepark 32a, Hagenberg, Austria; 5 cortEXplore GmbH, Industriezeile 35, 4020 Linz, Austria; 6 Johannes Kepler University Linz, Institute of Polymer Product Engineering, Altenberger Strasse 69, Linz, Austria; 7 R’n’B Consulting GmbH, Steingasse 6a, 4020 Linz, Austria; 8 Alpha Medical Concepts E.U., 4060 Leonding, Austria; 9 Eulerian-Solutions E.U. Leonfeldnerstraße 245, 4020 Linz, Austria; 10 Department of Neurosurgery and Clinical Neuroscience Center, University Hospital Zurich, Zurich, Switzerland

**Keywords:** Mixed-reality, Aneurysm clipping, Blood flow simulation, Validation

## Abstract

Nowadays, surgical treatment of cerebral aneurysms remains one of the most demanding disciplines in neurosurgery. The increasing shift toward endovascular interventions leads to a decline in open surgical cases. This fact leaves residents and young neurosurgeons with fewer training opportunities and limited to complex and high-risk aneurysms. There is a growing need for realistic simulation tools to enhance neurosurgical training and preoperative planning. We developed and externally validated a patient-specific mixed-reality simulator for cerebral aneurysm clipping, during the research project "Medical EDUcation in Surgical Aneurysm Clipping (MEDUSA)". Our approach combines physical phantoms of the skull and brain tissue with virtual intracranial blood vessels, including a virtual intracranial aneurysm. Real surgical instruments provide an immersive training environment featuring integrated blood flow simulation for evaluating clipping strategies. A life-sized skull with silicone brain lobes is mounted in a standard neurosurgical head clamp. Optical tracking synchronizes the position of a real clip applier and an emulated surgical microscope with the corresponding virtual environment, allowing true mixed-reality interaction. After aneurysm clipping, blood flow is automatically simulated to assess residual aneurysms or stenoses of the parental vessels. We conducted an external validation with 40 neurosurgeons at two international events. Participants completed a 32-item questionnaire evaluating face and content validity on a 5-point Likert scale. Participants' surgical experience ranged from novice to expert (> 15 years). Average ratings for simulator realism and educational value were high, with mean scores between 3.13 and 4.25. The highest ratings were for the blood flow simulation (4.25) and the simulator's potential for preoperative planning (4.20). Most participants agreed that the physical and virtual components were valuable and that the simulator should be integrated into neurosurgical training and standard surgical workflows. Our mixed-reality simulator achieved robust face and content validity among a diverse group of neurosurgeons. Combining real surgical instruments with a deformable virtual aneurysm model, including blood flow simulation, offers a high level of realism and immediate objective feedback.

**Supplementary Information:**

The online version contains supplementary material available at 10.1007/s10143-025-03846-x.

## Introduction

Surgical treatment of cerebral aneurysms remains one of the most demanding disciplines in neurosurgery [1, 2]. Over the last two decades, especially after the ISAT trial [3], there has been a significant shift towards endovascular techniques [4]. Surgical treatment is now often reserved for aneurysms with complex anatomical features or in cases where endovascular therapy cannot be performed safely or effectively. This paradigm shift has led to a challenging situation for residents and young neurosurgeons [2, 5], as they are increasingly confronted with aneurysms that eventually require a well-trained and experienced neurosurgeon. However, the decision between surgical and endovascular treatment depends on multiple factors, including aneurysm characteristics and patient-specific considerations such as age, comorbidities, frailty, and overall clinical condition [6].

In addition to aneurysm clipping, an exact surgical strategy should further consider the surrounding anatomical structures (aneurysm, brain tissue, skull), making preoperative planning and intraoperative decision-making highly demanding [2, 5, 7]. Therefore, these challenges underscore the need for innovative simulation strategies to optimize surgical education and operative procedure planning [1] in a safe and realistic environment.

Integrating simulation into neurosurgical education could provide residents with valuable hands-on experience and allow even experienced neurosurgeons to evaluate different operative strategies before entering the operating room. In recent years, significant advancements have been made in vascular neurosurgery research, leading to various simulation models, including cadaver models, virtual reality systems, and 3D physical models using e.g. 3D printing technologies [7–16].

We developed a patient-specific clipping simulator within the project MEDUSA (Medical EDUcation in Surgical Aneurysm clipping) using a mixed-reality approach that combines physical (skull, brain tissue) and virtual (intracranial blood vessel and aneurysms) elements. The simulation is performed with original surgical instruments, while the surgical procedure is implemented through modular components. A virtual blood flow simulation assesses the effectiveness of aneurysm clipping by identifying residual aneurysms or potential vessel occlusions.

We further externally validated our clipping module during an international aneurysm clipping course (Zürich aneurysm clipping course, Tübingen, Germany, 2024) and a conference (11th annual EANS vascular section meeting, Marseilles, France, 2024) with 40 neurosurgeons.

## Material and methods

### Simulator development

The simulator presented in this study is an advancement of an earlier prototype described previously [17]. Between 2019 and 2024, it was further developed into a hybrid aneurysm clipping simulator as part of a joint research project funded by the government of Upper Austria involving academic and industry partners. The goal was to create a simulation environment that resembles the real situation in the operating room as closely as possible, including a simulated surgical microscope and the usage of real instruments and phantoms with tissue-like haptic properties based on patient data. Virtual and physical parts are synchronized by a customized optical tracking system (cortEXplore GmbH), yielding a hybrid simulation system.

**Fig. 1:**
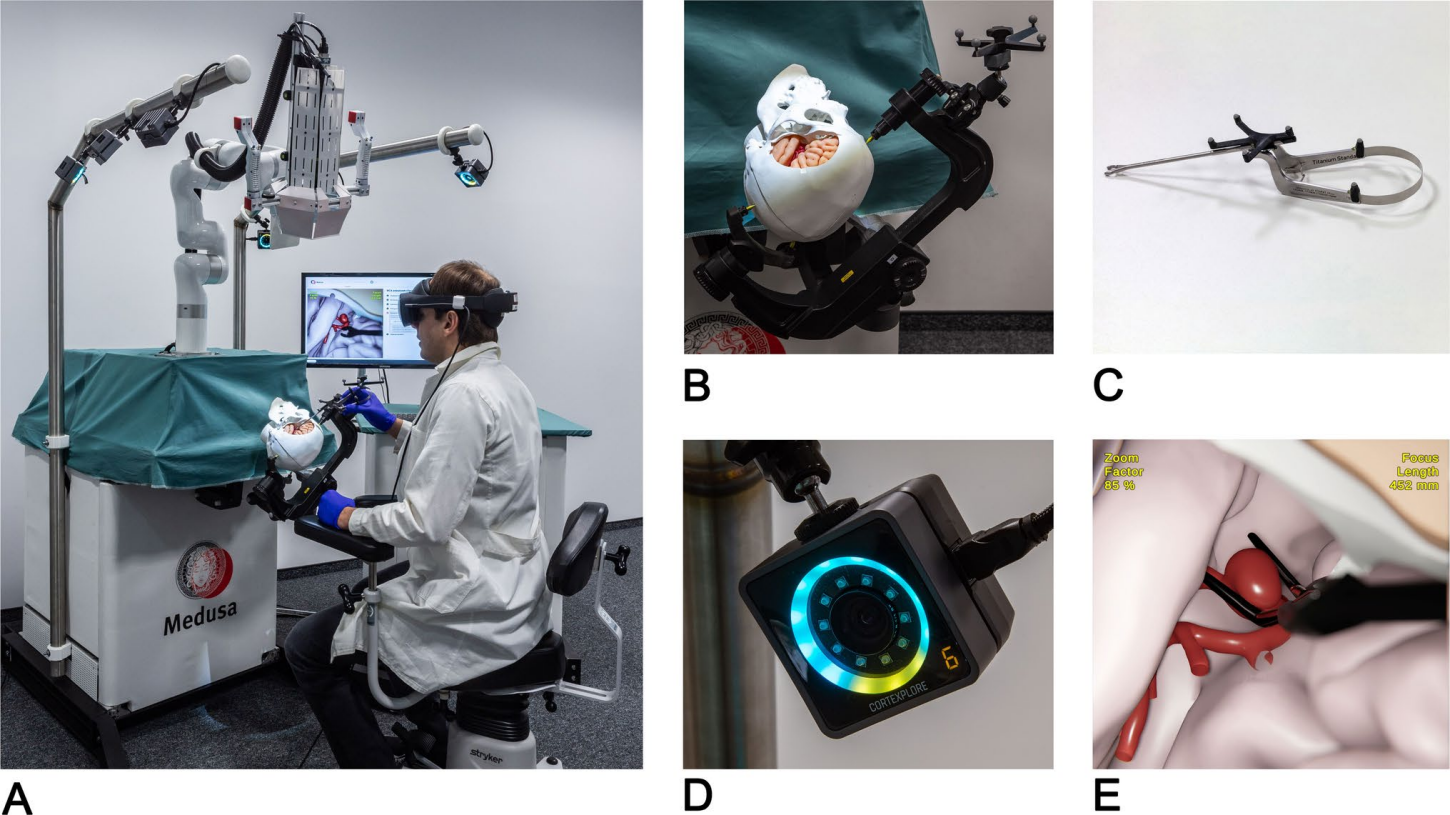
Components of the Medusa simulator. (**A**) mixed reality simulator; (**B**) detailed view of patient phantom showing the clamped head and artificial skull and brain; (**C**) modified clipping forceps with reflective markers; (**D**) optical tracking camera; (**E**) virtual view of the operating field (middle cerebral artery aneurysm)

**Fig. 2:**
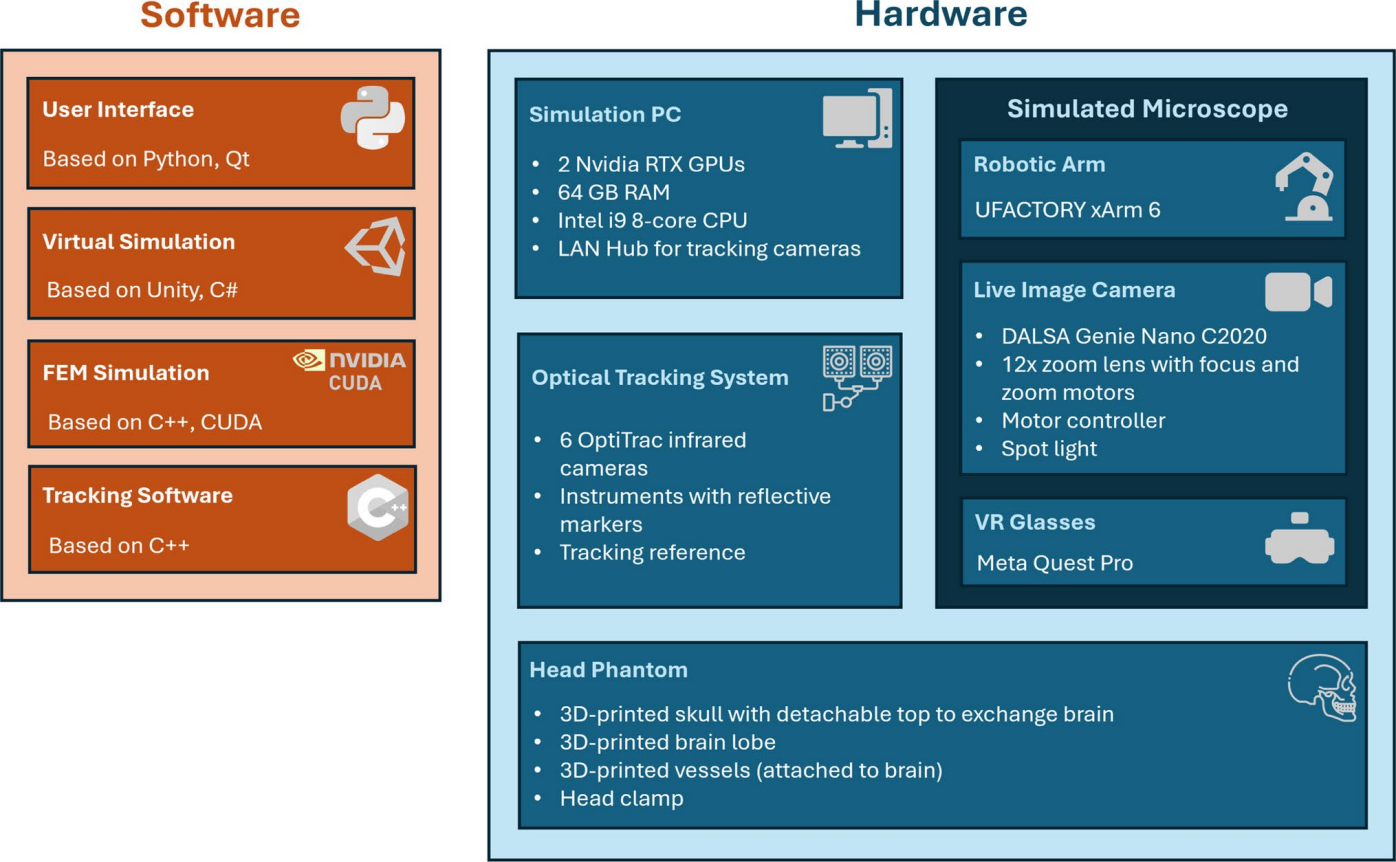
Schematic overview of the hardware and software components used in the Medusa simulator

The complete setup of the simulator with a neurosurgeon performing a training session is illustrated in Fig. 1A. The system consists of the central part on the left containing a personal computer, an optical tracking system, a head clamp with the head phantom, and the emulated operating microscope. A schematic overview of the hardware and software components of the simulator, including the integration of virtual simulation, tracking, and physical elements such as the phantom and simulated microscope, is provided in Fig. 2 to complement the photographic view shown in Fig. 1A. The latter is a camera attached to a robotic arm that can be moved in zero-gravity mode to define the point-of-view for the virtual scene. The zoom step and focus plane can be adjusted with buttons on the microscope's handles. The neurosurgeon sits on a real surgical chair, observing the operating field through virtual reality glasses (Meta Quest Pro, Meta Platforms, Inc., California, United States). Simultaneously, the same view is presented as part of the simulation software user interface on an extra monitor. Thus, additional persons can follow the training, discuss with the trainee, and perform further actions in the software (such as choosing a different clip type) via mouse and keyboard.

Figure 1B gives a detailed view of the artificial skull and brain, fixed in a standard surgical head clamp. The skull can be freely positioned since the correct orientation of the phantom is an essential part of the training. The 3D-printed rigid skull comes with a predefined pterional craniotomy, which can be decreased in size by inserting different stencils. Inside the skull, a phantom of the left-brain hemisphere made of silicone (ECOFLEX 10, Smooth-On, Inc., Pennsylvania, United States) is situated, including the temporal and frontal lobe and the Sylvian fissure in between. Using a haptic model for the brain ensures that the possible instrument trajectories (position and orientation) are restricted in the same way as in reality. 3D-printed blood vessels are also present but serve only for visual guidance. As our focus was to minimize distraction and ensure a realistic simulation environment, we opted for a real clip applier instead of e.g. a haptic input device.

The applier is equipped with reflective markers (see Fig. 1C), which are tracked by six infrared cameras (Fig. 1D and A), providing the instrument position and orientation in space relative to a fixed reference (top right in Fig. 1B) with an accuracy of up to 0.1 mm. To map the coordinates to the 3D models of the patient, thus achieving an exact correspondence between the real and virtual world, a registration procedure has to be performed at least once after the skull is fixed in the clamp. Using landmarks on the surface of the phantoms, both a rigid and nonrigid registration step is carried out, accounting for a possible deformation of the soft tissue concerning the 3D model. In the same way, the view in the virtual world is adjusted according to the markers attached to the microscope.

Finally, Fig. 1E shows the virtual view of the operating field presented to the neurosurgeon in the VR glasses. The instruments and anatomic structures, including a detailed model of the aneurysm wall (middle cerebral artery aneurysm), are rendered realistically, including shadows, metallic reflections, and simulated depth of field. The clip applier has one specific clip from an extendable clip library (more than 50 different clip types; Aesculap AG, Tuttlingen, Germany), which can be changed as needed. While the clip applier is present in both realities (physical and virtual), the clip itself is virtual only. The clip interacts with the virtual aneurysm walls, which react to collisions and deform accordingly. A finite-element-based physical elasticity model calculates the wall deformation in real time, providing immediate visual feedback.

**Fig. 3:**
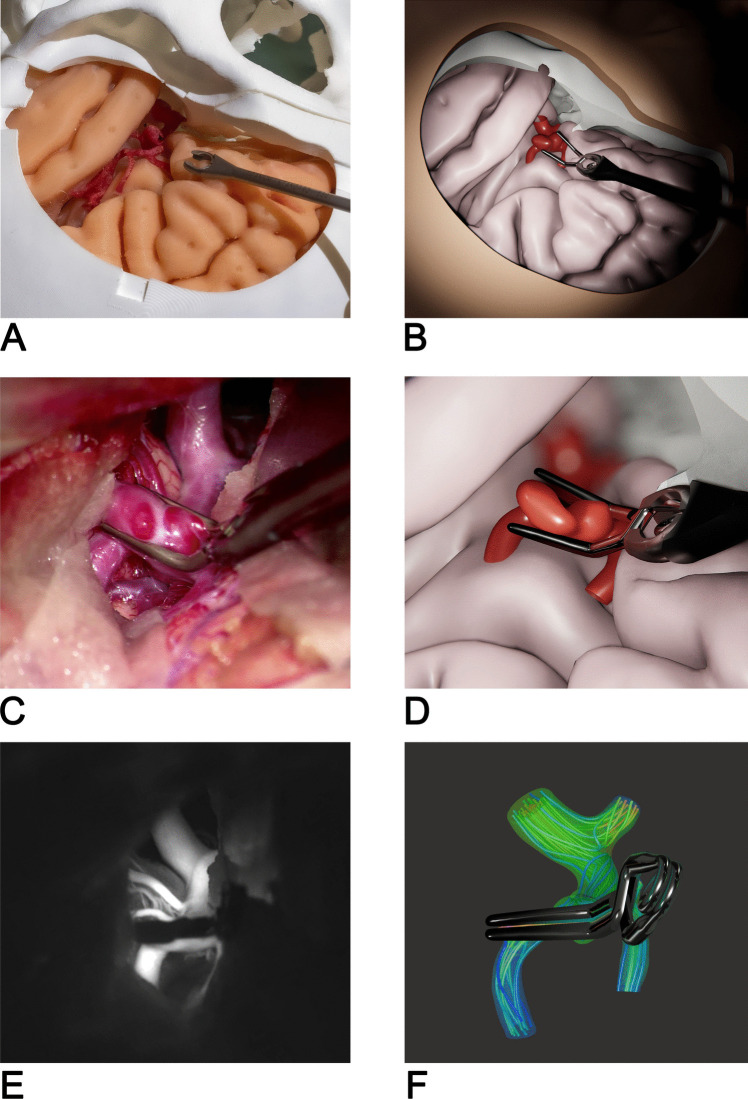
Comparison between physical and virtual world. (**A**) View of the physical operating field; (**B**) View of the virtual operating field showing the accurate correspondence between the two. Note that the clip only exists in the virtual world; (**C**) Scene from a real middle cerebral artery aneurysm clipping surgery; (**D**) Virtual clipping of the same aneurysm reconstructed from image data of the patient; (**E**) ICG angiography showing blood perfusion after clipping; (**F**) visualization of streamlines and pressure distribution obtained from simulation of blood flow through the clipped aneurysm model

Figure 3 demonstrates our simulator's hybrid nature and the advantages of this approach.

The 3D-printed parts (Fig. 3A) ensure a realistic haptic perception during training, by restricting trajectories to achievable real-world scenarios. On the other hand, in the virtual simulation part (Fig. 3B), patient-specific models can be created within a short period, allowing non-destructive interactions and unlimited training iterations. An accurate real-time synchronization between the two realities is essential for a realistic simulation experience. Figure 3C and D, respectively, compare a microscopic view of a real clipping surgery and the virtual equivalent using the geometry obtained from the corresponding image data. Figure 3E shows an ICG-angiographic view of the clipped aneurysm, demonstrating the absence of residual blood flow. In the virtual simulation, a detailed blood flow calculation (Fig. 3F) allows an assessment of the chosen clipping strategy by evaluating occlusion and possible induced stenosis of the parent artery.

**Fig. 4:**
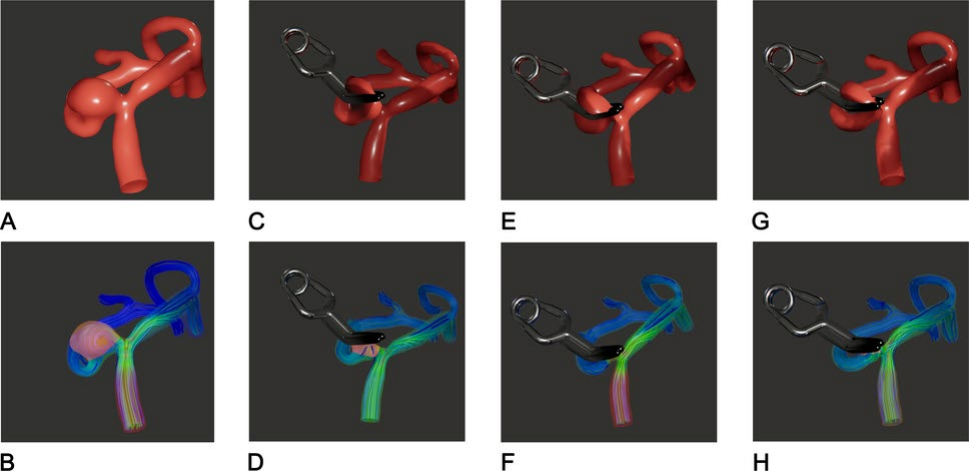
Virtual blood flow simulation to evaluate a specific clipping strategy. The upper row shows the clip–aneurysm complex without blood flow simulation, while the lower row presents the corresponding blood flow simulations. Different scenarios are illustrated: pre-clipping (**A**, **B**), residual aneurysm (**C**, **D**), critical M2 stenosis (**E**, **F**), and a regular post-virtual clipping result (**G**, **H**)

Figure 4 illustrates different scenarios before and after clip application in a middle cerebral artery aneurysm (Fig. 4A and B): a residual aneurysm (Fig. 4C and D), a critical M2 branch stenosis (Fig. 4E and F), or a regular result (Fig. 4G and H).

**Fig. 5:**
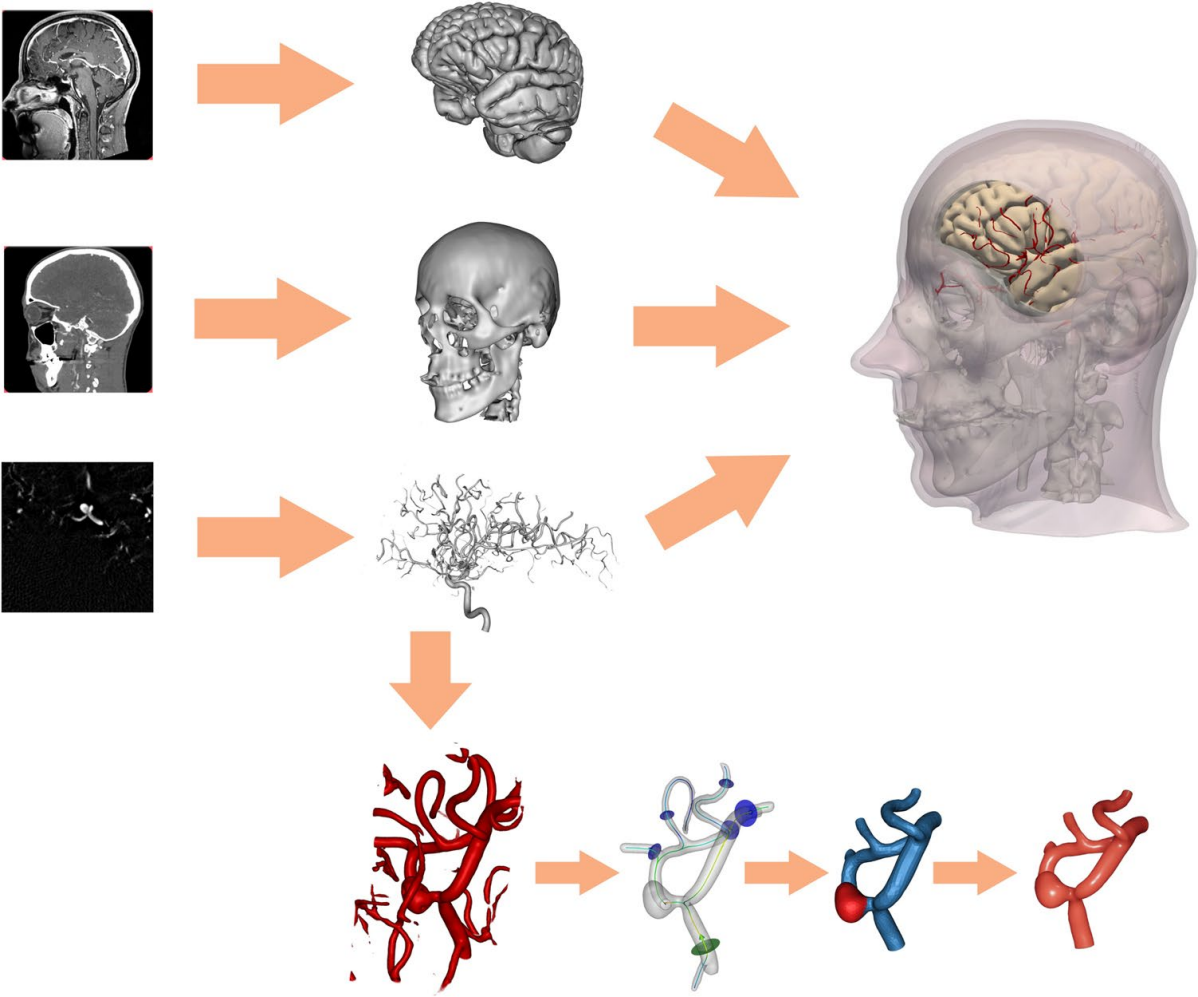
Schematic overview of the processing pipeline for obtaining patient-specific 3D models from image data. Typically, the skull is extracted from CT, the brain is extracted from MRI and the blood vessels are extracted from DSA scans. The deformable arterial wall in the region close to the aneurysm requires additional steps, such as the definition of in- and outflow planes and separation of the aneurysm sac

To facilitate the integration of patient-specific geometries into the simulator, a dedicated tool was developed to extract anatomical structures from multimodal image data consisting of computed tomography, magnetic resonance imaging, and digital subtraction angiography, all acquired from the same patient. The tool includes a streamlined processing pipeline that segments the target anatomical structures, performs co-registration to align the structures, and constructs composite 3D models. This process combines AI-based and traditional image processing methods, some of which were specifically developed and disseminated within the scope of the project [18–22]. In addition to anatomical structure extraction, the tool facilitates the annotation of interventional and simulation-specific parameters such as the definition of the target aneurysm and the placement of the craniotomy. It was optimized to enable the extraction and processing of all data required for simulation in less than 15 min. Figure 5 presents a schematic overview of the processing pipeline.

Virtual clipping and assessment of the procedure using blood flow simulation are also illustrated in Video 1.

### Validation study

The simulator was validated during the Zurich Aneurysm Clipping Course (Tübingen 2024; n = 22) and the 11th Annual EANS Vascular Section Meeting (Marseilles 2024; n = 18), using a questionnaire with 32 questions addressing the face and content validity of the simulator. Questions were rated using a 5-point Likert scale, ranging from "strongly disagree" (score 1) to "strongly agree" (score 5), and free-text responses were also possible. Each participant's years of surgical experience, number of clipped aneurysms, number of brain tumor surgeries, and number of performed procedures involving the splitting of the Sylvian fissure were recorded. Based on these numbers, participants were classified as novices (≤ 20), intermediate-level neurosurgeons (> 20 and < 100), or experts (> = 100). In addition to descriptive statistics, the scores were tested for statistical differences between experts, intermediates, and novices. The examined data was tested for normal distribution with the Shapiro–Wilk test for small sample sizes. For normally distributed results, the Levene test was used to test for variance homogeneity. The Mann–Whitney U test was utilized for non-normally distributed data or data with inhomogeneous variances to detect differences between groups. For normally distributed results with homogeneous variances, Student's T-test for independent variables was used to detect differences between groups. For all tests, a p-value of 0.05 or less was considered significant.

The local ethics committee (Ethikkommission der medizinischen Fakultät der Johannes Kepler Universität; EK Nr:1082/2023) approved the validation study design. All procedures performed were in accordance with the ethical standards of the institutional and national research committees, as well as with the 1964 Helsinki Declaration and its later amendments or comparable ethical standards. All participants took part voluntarily, and informed consent was waived.

## Results

**Fig. 6:**
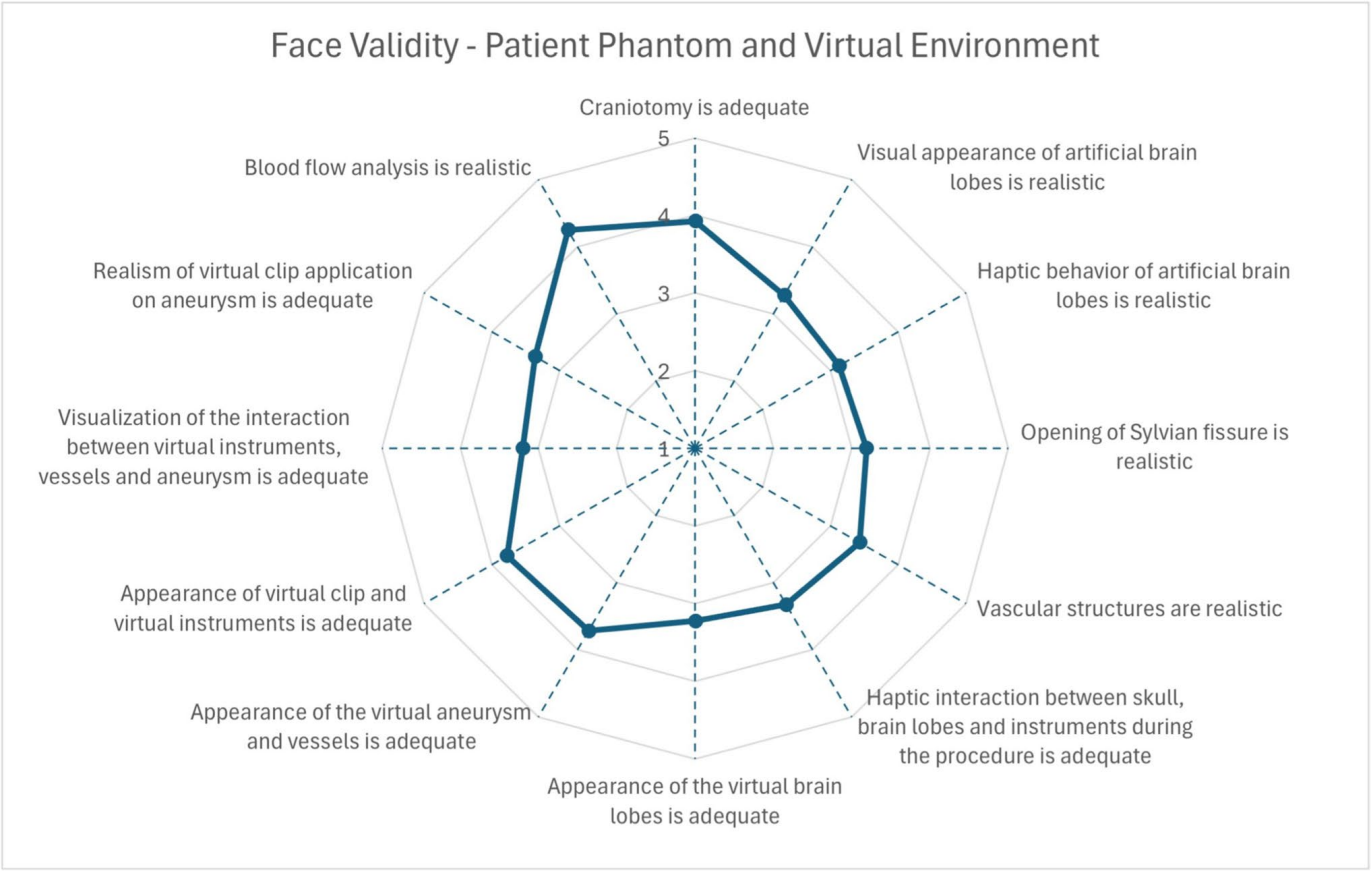
Mean scores for face validity of patient phantom and virtual environment

**Fig. 7:**
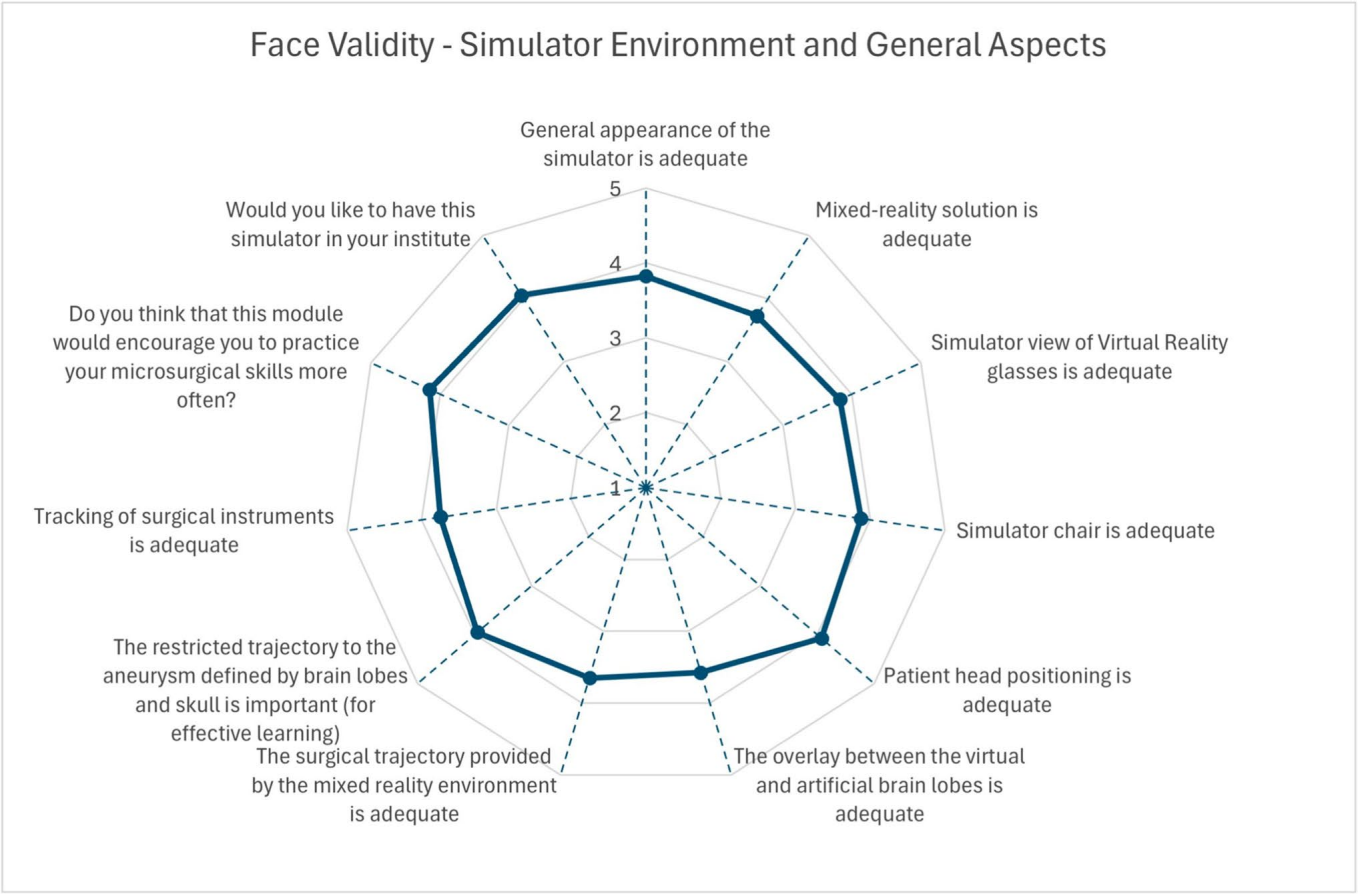
Mean scores for face validity of simulator environment and general aspects

**Fig. 8:**
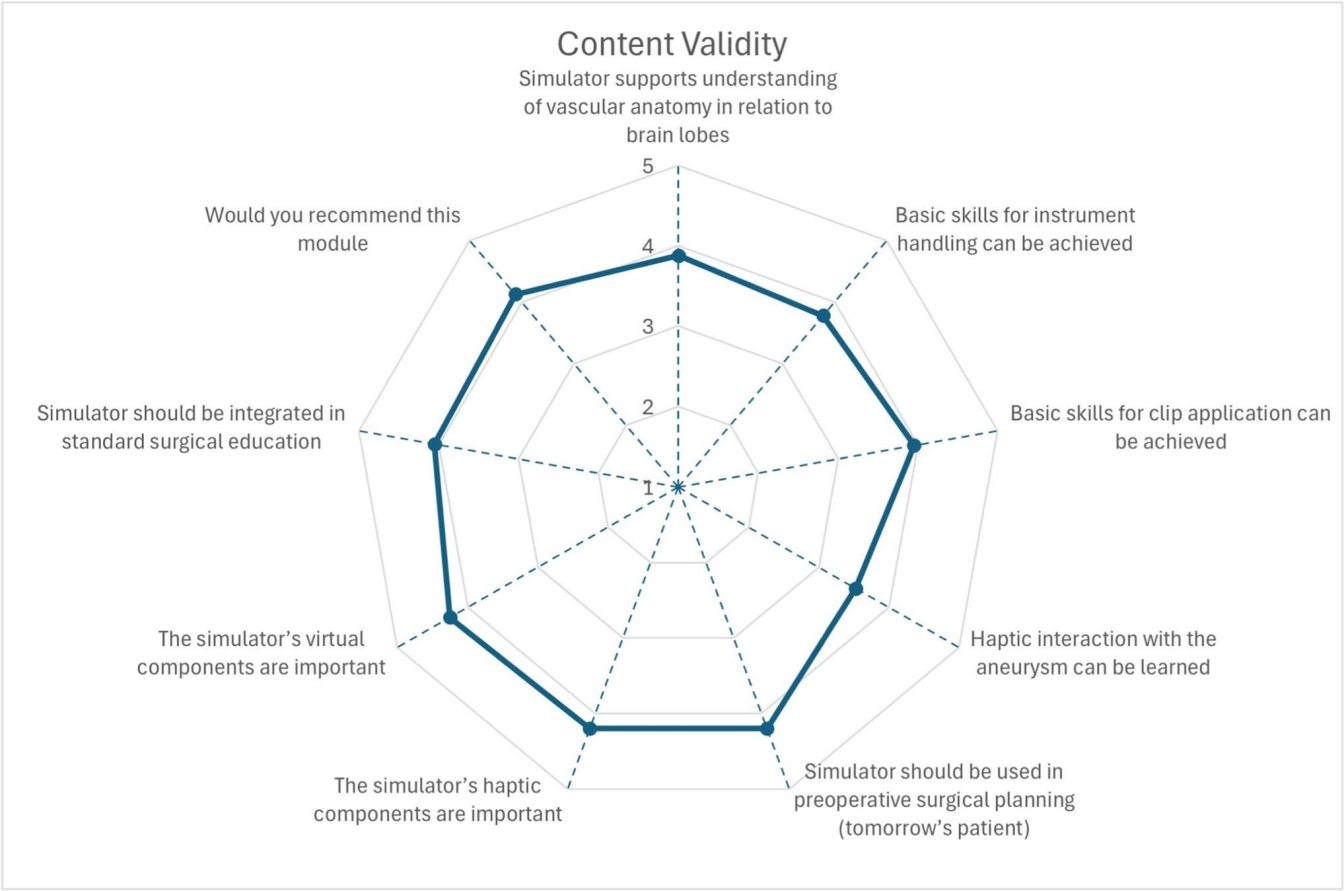
Mean scores for content validity of the mixed reality clipping simulator

All participants (n = 40) received an introduction to the simulator before evaluating the simulation. There were 11 novices, 17 intermediate-level neurosurgeons, and 12 experts. Mean surgical experience ranged from less than 1 year to 38 years, with expert neurosurgeons having at least 15 years of experience. The mean scores for questions concerning the face and content validity of the simulator are shown in Figs. 6, 7 and 8, which ranged from 3.13 to 4.25.

Most participants agreed that the blood flow simulation is realistic (mean score of 4.25) and that the simulator should be integrated into preoperative planning (mean score of 4.20) and standard surgical education (mean score of 4.05). Most users considered the physical (mean score of 4.20) and virtual (mean score of 4.25) components important. We achieved good results for the appearance of both virtual and physical components (mean scores ranging from 3.28 to 3.78). The study participants perceive the haptic interaction between the instrument and artificial anatomy as realistic (mean scores between 3.13 and 3.93). Statistically significant differences were found for the haptic interaction between instruments and artificial anatomy (experts: 2.58 ± 0.76, intermediates: 3.71 ± 0.89, novices: 3.54 ± 0.89) with p-values 0.002 for experts vs. intermediates and 0.015 for experts vs. novices Consequently, experts rated that the haptic interaction with the aneurysm can be learned with 2.92 ± 1.11, where the rates of intermediates and novices were 3.65 ± 1.19 and 4.00 ± 0.74, respectively. Statistically significant differences were found here between experts and novices (p-value: 0.016). Most neurosurgeons agreed that the surgical trajectory to the aneurysm, defined by brain lobes and the skull, is important (mean score of 3.95). The mixed reality environment and overlay between virtual and physical brain lobes were rated with mean scores of 3.65 and 3.58, respectively. Most participants agreed that basic skills for instrument handling (mean score of 3.78) and clip application (mean score of 3.95) can be achieved using this simulator.

## Discussion

In this study, we successfully developed a novel patient-specific mixed-reality simulator for aneurysm clipping at the Department of Neurosurgery, Kepler University Hospital in Linz, from 2019–2024. This approach was externally validated with 40 neurosurgeons.

Neurosurgical operations are highly demanding procedures in eloquent operative regions with little margin for error [23]. Therefore, strategies to improve the quality of neurosurgical education in an effective and safe environment are highly warranted. Nowadays, neurosurgeons are confronted with working time regulations and increasing case complexity in low-case situations, which might impact the learning curve [24, 25]. In a systematic review, a warm-up effect was demonstrated for the laparoscopic performance of general surgeons in five out of six studies [26]. Psychomotor and cognitive skills are significantly improved in all groups with differing experience levels, even after a short-term warm-up of 15 min [27].

In general surgery, simulation-based training has recently led to significant modifications regarding the structure and content of surgical curricula, and specific training programs have been developed [28, 29]. In a meta-analysis it could be demonstrated that laparoscopic cholecystectomies might be performed more effectively with reduced technical errors and faster completion times by virtual-reality-trained surgeons [30].

Similarly, directors of 99 U.S. Neurosurgery programs were asked via a 14-item questionnaire how simulation could be implemented in residency programs. Seventy-two and 74% of responding directors believed that simulations would improve patient outcomes or should be combined with standard education, respectively [31].

Virtual reality has already been used to plan aneurysm clipping surgery by integrating virtual head positioning, craniotomy, and surgical trajectory, as well as determining the angle of clip application with diverging results. Yet, realistic aneurysm deformation, opening or closing of the clip, and blood flow simulation after training were not integrated in previous studies [32, 33].

Several studies have implemented haptic feedback in their virtual simulations [8, 14, 17]. Using the Immersive-Touch platform, a standard middle cerebral artery aneurysm could be clipped with deformation of the aneurysm using two haptic input devices. In our predecessor project, "Virtual Aneurysm," [17], we improved these approaches and primarily focused on cerebral aneurysm clipping. We used an original clipping forceps mounted on a haptic input device and implemented a blood flow simulation for training assessment.

Recently, physical models of cerebrovascular anatomy were significantly refined using 3D printing and casting technology, creating realistic and soft elastic vascular structures combined with skull and brain models, allowing the aneurysm neck to be occluded during simulation [7, 9, 12, 34, 35]. Additionally, models with hollow elastic vessels were improved by simulating or analyzing blood flow [7, 11, 36, 37].

In this study, we have chosen a patient-specific mixed-reality solution, where the approach to the aneurysm is provided through physical simulation, while the clipping procedure and assessment using blood flow simulation are presented in virtual reality. This approach has several advantages and offers the possibility of surgeon-specific training structured into modules. Although we have only validated the clipping module in this study, inexperienced residents can simulate the entire chain of the surgical procedure: head positioning, planning of the surgical trajectory, craniotomy, splitting of Sylvian fissure, clipping, and training assessment. However, an expert may only want to practice clipping strategies in virtual reality before surgery.

We have additionally developed a prototype for splitting an artificial Sylvian fissure. This component has not yet been validated, which we plan to address in subsequent studies. However, this surgical step might be challenging, especially in certain situations (e.g., after subarachnoid hemorrhage). Therefore, the availability of a simulator for training and preparation might be particularly important.

Furthermore, in emergencies, we could quickly set up the virtual components and simulate the clipping of a ruptured aneurysm within 15 min. Consequently, our approach could be rapidly and realistically implemented in a clinical setting, offering a clear advantage over 3D-printed models. To train intraoperative complication management, we have implemented a simulated intraoperative virtual aneurysm rupture. This situation can be managed by placing a temporary clip on the M1 segment, followed by definitive clipping.

For the training of different clipping strategies, the optimal surgical trajectory might be essential. These trajectories are constrained by realistic boundaries provided by physical brain lobes and the rigid skull, typically unavailable in pure virtual simulators [17].

Achieving precise temporal and spatial synchronization of the physical instruments and phantoms with their virtual counterparts is technically demanding, requiring accurate and reliable registration of all physical components.

This determines the accurate visualization of anatomical relationships between brain lobes and blood vessels and affects the haptic feedback during interactions between instruments and the simulator's physical components. It is important to note that the vascular structures embedded in the physical brain model are purely visual guides and do not interact with the virtual clip or the simulated blood flow. Consequently, a potential misalignment between the physical and virtual vasculature has no impact on the clipping procedure itself or on the subsequent assessment of blood flow. At most, users may perceive a minor visual offset when switching focus between the physical and virtual elements, which does not affect training outcomes or the evaluation of clipping strategies. Since the physical vessel structures are purely static and non-interactive, any tactile feedback from unintentionally touching them does not influence the virtual clipping procedure or blood flow assessment; potential sensory discrepancies are therefore negligible and were not reported as an issue during validation.

So far, only a few studies have introduced mixed reality, hybrid, or augmented reality solutions in aneurysm clipping simulation, with different applications and varying degrees of complexity [38, 7].

Dodier et al. presented an elegant approach using patient-specific aneurysm simulators featuring hollow, perfused, and elastic aneurysm models created with 3D printing technology. In addition to this physical simulation, these holographic aneurysm models can be integrated into an augmented reality head-up display, allowing virtual clipping [36]. Teodoro-Vite et al. developed a hybrid simulation of a physical skull combined with virtual brain lobes, vessels, and aneurysms. Clipping is performed using haptic input devices.

Unlike our approach, these studies did not integrate virtual blood flow simulation, and virtual clipping was not performed using original clipping forceps [15, 36, 38]. Training on realistic instruments is especially essential in surgical education. Moreover, adapting these simulation systems to new patient-specific geometries can be challenging due to the complexities involved in image segmentation and modeling [15]. Most importantly, because of the implemented blood flow simulation, an immediate and objective evaluation of the simulation is possible. This allows the testing of different clip strategies and facilitates the visualization of critical stenoses or residual aneurysms. Overall, in our validation, the blood flow simulation was the highest-rated component (mean score 4.25), underscoring its significance.

In this study, we successfully validated face and content validity, with scores ranging from 3.13 to 4.25. We subdivided the participants into novices, intermediate-level neurosurgeons, and experts with extensive experience. Experts had more than 15 years of surgical experience. Other studies have defined experts as those who have clipped fewer aneurysms or were even in late residency [11, 38]. Furthermore, we validated our approach during an international workshop and a congress to avoid biases that might have arisen if we had only performed an internal validation.

Most participants strongly agreed that our approach could teach basic skills for clip application (mean score 3.95) and that it should be integrated into preoperative planning and standard surgical education (mean scores > 4). These results align with other studies addressing aneurysm clipping simulation [7, 8, 17]. In the next step, we are developing a didactic framework to integrate this type of simulation into educational programs at hospitals and during clipping workshops. Moreover, we have already demonstrated that such an approach may be reliably incorporated into well-established courses (e.g., the Zürich Aneurysm Clipping Course).

Although various simulation methods [7] (ex vivo, virtual reality, or 3D-printed models) are available, participants still indicated that both virtual and physical components are important (mean score > 4.2). The surgical trajectory offered by the mixed reality environment is particularly significant (mean score 3.65), as purely virtual simulations might permit unrealistic clip trajectories. For this reason, the overlay between virtual and artificial brain lobes provided by optical neuronavigation is essential. Although we obtained acceptable results regarding these technical challenges, we are working on a further improvement. Mapping the 3D-printed brain lobes to the virtual models is complicated because the soft material deforms under the influence of gravity and the interaction with the instrument. Furthermore, the position and orientation inside the skull can deviate from those obtained from the medical image data.

We achieved acceptable results concerning the appearance and realism of different virtual and artificial components. However, these are aspects that warrant improvement in future research.

A limitation of this study is that we have not validated construct (the ability of the simulator to distinguish between users of different expertise levels) and predictive (whether training on the simulator translates into improved performance in real-life surgery) validity. Further, prospective studies for evaluating the long-term educational and clinical impact of simulation-based training are planned. Additionally, we need to extend our validation by including anatomically realistic arachnoid membranes to model the splitting of the Sylvian fissure. Furthermore, we have not yet simulated cerebrospinal fluid dynamics. The aneurysm wall is simulated using homogeneous material properties. No perforators or variable vascular wall thickness, including complex aneurysms such as intraluminal thrombus or calcifications, have been integrated. Patient-specific biomechanical characteristics would allow for a more realistic simulation.

Currently this prototype allows for real-time interaction with patient-specific virtual aneurysms. To simulate bimanual surgical techniques that are essential for dissection and clip placement we need to incorporate the stable simulation and tracking of a second instrument (e.g., suction or dissector) to enhance the realism of the training scenario further. Also, we plan to simulate the application of multiple clips to an aneurysm, including the handling of collisions between separate clip models. The library of available clip geometries will also be extended. At the moment, interaction between instruments and the virtual brain lobes is not simulated, but is currently in development.

## Conclusion

This study demonstrates the successful development and external validation of a patient-specific mixed-reality simulator for intracranial aneurysm clipping. The system delivers a highly realistic and objective training environment by combining real instruments, a silicone brain in a physical skull phantom, and virtual 3D aneurysm models—supplemented with real-time blood flow simulation. Future work will focus on additional instruments and further patient-specific features to enhance neurosurgical education and preoperative planning.

## Supplementary Information

Below is the link to the electronic supplementary material.

Supplementary file1 (MP4 7781 KB)

## Data Availability

Data will be made available from the corresponding author upon reasonable request.

## References

[1] Rehder R et al (2016) The role of simulation in neurosurgery. Childs Nerv Syst 32:43–54 26438547 10.1007/s00381-015-2923-z

[2] Scullen T et al (2024) Training cerebrovascular and neuroendovascular surgery residents: a systematic literature review and recommendations. Ochsner J 24:36–46 38510222 10.31486/toj.23.0118 PMC10949058

[3] Gnanalingham KK, Apostolopoulos V, Barazi S, O’Neill K (2006) The impact of the international subarachnoid aneurysm trial (ISAT) on the management of aneurysmal subarachnoid haemorrhage in a neurosurgical unit in the UK. Clin Neurol Neurosurg 108:117–123 16364540 10.1016/j.clineuro.2005.11.001

[4] Calvanese F et al (2024) Changes in treatment of intracranial aneurysms during the last decade in a large European neurovascular center. Acta Neurochir (Wien) 166:173 38594469 10.1007/s00701-024-06064-4 PMC11004042

[5] Wurm G, Lehner M, Tomancok B, Kleiser R, Nussbaumer K (2011) Cerebrovascular biomodeling for aneurysm surgery: simulation-based training by means of rapid prototyping technologies. Surg Innov 18:294–306 21307017 10.1177/1553350610395031

[6] Keegan A, Hicks CW (2022) Surgical decision-making and outcomes in open versus endovascular repair for various vascular diseases. Anesthesiol Clin 40:627–644 36328619 10.1016/j.anclin.2022.08.008 PMC9833286

[7] Joseph FJ, Vanluchene HER, Bervini D (2023) Simulation training approaches in intracranial aneurysm surgery—a systematic review. Neurosurg Rev 46:101 37131015 10.1007/s10143-023-01995-5 PMC10154262

[8] Alaraj A et al (2015) Virtual reality cerebral aneurysm clipping simulation with real-time haptic feedback. Neurosurgery 11(Suppl 2):52–58 25599200 10.1227/NEU.0000000000000583 PMC4340784

[9] Belykh E et al (2021) Novel system of simulation models for aneurysm clipping training: description of models and assessment of face, content, and construct validity. Oper Neurosurg 21:558–569 34662910 10.1093/ons/opab357

[10] Colombo E et al (2024) Intensive 2-days training on perfused human placenta for microvascular anastomoses. Acta Neurochir (Wien) 166:459 39545974 10.1007/s00701-024-06286-6 PMC11568009

[11] Joseph FJ, Weber S, Raabe A, Bervini D (2020) Neurosurgical simulator for training aneurysm microsurgery—a user suitability study involving neurosurgeons and residents. Acta Neurochir (Wien) 162:2313–2321 32780255 10.1007/s00701-020-04522-3 PMC7496061

[12] Mashiko T et al (2015) Development of three-dimensional hollow elastic model for cerebral aneurysm clipping simulation enabling rapid and low cost prototyping. World Neurosurg 83:351–361 24141000 10.1016/j.wneu.2013.10.032

[13] Oliveira MM et al (2019) Quality assurance during brain aneurysm microsurgery—operative error teaching. World Neurosurg 130:e112–e116 31176838 10.1016/j.wneu.2019.05.262

[14] Shono N et al (2018) Microsurgery simulator of cerebral aneurysm clipping with interactive cerebral deformation featuring a virtual arachnoid. Oper Neurosurg 14:579–589 28973685 10.1093/ons/opx155

[15] Vite ST, Velasco CD, Valencia AFH, Lomelí JSP, Castañeda MÁP (2018) Virtual simulation of brain sylvian fissure exploration and aneurysm clipping with haptic feedback for neurosurgical training. In: Augmented Reality, Virtual Reality, and Computer Graphics. Springer, Cham, pp 230–238. 10.1007/978-3-319-95282-6_17

[16] Aboud E et al (2015) Live cadavers for training in the management of intraoperative aneurysmal rupture. J Neurosurg 123:1339–1346 26140492 10.3171/2014.12.JNS141551

[17] Gmeiner M et al (2018) Virtual cerebral aneurysm clipping with real-time haptic force feedback in neurosurgical education. World Neurosurg 112:313–323 29337170 10.1016/j.wneu.2018.01.042

[18] Sabrowsky-Hirsch B, Schenkenfelder B, Klug C, Reishofer G, Scharinger J (2022) Deformable Registration of Low-overlapping Medical Images. In: 2022 21st IEEE international conference on machine learning and applications, ICMLA, pp 940–945. 10.1109/ICMLA55696.2022.00157

[19] Sabrowsky-Hirsch B, Moser P, Thumfart S, Scharinger J (2024) Segmentation and anatomical annotation of cerebral arteries in non-angiographic MRI. In: Proceedings of the 2023 6th international conference on digital medicine and image processing. Association for Computing Machinery, New York, NY, USA, pp 74–81. 10.1145/3637684.363769

[20] Sabrowsky-Hirsch B, Alshenoudy A, Thumfart S, Giretzlehner M, Scharinger J (2024) Brain Artery Segmentation for Structural MRI. In: openreview.net, Paris, France

[21] Sabrowsky-Hirsch B et al (2024) Robust multi-modal registration of cerebral vasculature. In: Medical image understanding and analysis. Yap MH, Kendrick C, Behera A, Cootes T, Zwiggelaar R eds. Springer Nature Switzerland, Cham, 2024, pp 3–18. 10.1007/978-3-031-66955-2_1

[22] Alshenoudy A, Sabrowsky-Hirsch B, Scharinger J, Thumfart S, Giretzlehner M (2024) Towards segmenting cerebral arteries from structural MRI. In: Medical image understanding and analysis. Yap MH, Kendrick C, Behera A, Cootes T, Zwiggelaa R eds. Springer Nature Switzerland, Cham, pp 19–33. 10.1007/978-3-031-66955-2_2

[23] Davids J et al (2021) Simulation for skills training in neurosurgery: a systematic review, meta-analysis, and analysis of progressive scholarly acceptance. Neurosurg Rev 44:1853–1867 32944808 10.1007/s10143-020-01378-0 PMC8338820

[24] Aggarwal R et al (2010) Training and simulation for patient safety. Qual Saf Health Care 19:i34–i43 20693215 10.1136/qshc.2009.038562

[25] Reznick RK (2006) Teaching surgical skills — changes in the wind. N Engl J Med. 10.1056/NEJMra054785 17182991 10.1056/NEJMra054785

[26] Abdalla G et al (2015) The effect of warm-up on surgical performance: a systematic review. Surg Endosc 29:1259–1269 25149639 10.1007/s00464-014-3811-4

[27] Kahol K, Satava RM, Ferrara J, Smith ML (2009) Effect of Short-Term Pretrial Practice on Surgical Proficiency in Simulated Environments: A Randomized Trial of the “Preoperative Warm-Up” Effect. J Am Coll Surg 208:255–268 19228538 10.1016/j.jamcollsurg.2008.09.029

[28] Lowry B, Johnson GGRJ, Vergis A (2022) Merged virtual reality teaching of the fundamentals of laparoscopic surgery: a randomized controlled trial. Surg Endosc 36:6368–6376 34981231 10.1007/s00464-021-08939-4 PMC8722746

[29] Shahrezaei A, Sohani M, Taherkhani S, Zarghami SY (2024) The impact of surgical simulation and training technologies on general surgery education. BMC Med Educ 24:1297 39538209 10.1186/s12909-024-06299-w PMC11558898

[30] Humm G et al (2022) The impact of virtual reality simulation training on operative performance in laparoscopic cholecystectomy: meta-analysis of randomized clinical trials. BJS Open 6:zrac086 35849132 10.1093/bjsopen/zrac086 PMC9291386

[31] Ganju A et al (2013) The role of simulation in neurosurgical education: a survey of 99 United States neurosurgery program directors. World Neurosurg 80:e1–e8 23182732 10.1016/j.wneu.2012.11.066

[32] Steineke TC, Barbery D (2021) Microsurgical clipping of middle cerebral artery aneurysms: preoperative planning using virtual reality to reduce procedure time. Neurosurg Focus 51:E12 34333481 10.3171/2021.5.FOCUS21238

[33] Wong GKC, Zhu CXL, Ahuja AT, Poon WS (2007) Craniotomy and clipping of intracranial aneurysm in a stereoscopic virtual reality environment. Neurosurgery 61:564–568, discussion 568–569 10.1227/01.NEU.0000290904.46061.0D 17881970

[34] Kimura T et al (2009) Simulation of and training for cerebral aneurysm clipping with 3-dimensional models. Neurosurgery 65:719–726 19834377 10.1227/01.NEU.0000354350.88899.07

[35] Petrone S et al (2022) Virtual-augmented reality and life-like neurosurgical simulator for training: first evaluation of a hands-on experience for residents. Front Surg 9:862948 35662818 10.3389/fsurg.2022.862948 PMC9160654

[36] Dodier P et al (2024) An evaluation of physical and augmented patient-specific intracranial aneurysm simulators on microsurgical clipping performance and skills: a randomized controlled study. Neurosurg Focus 56:E9 38163349 10.3171/2023.10.FOCUS23640

[37] Liu Y et al (2017) Fabrication of cerebral aneurysm simulator with a desktop 3D printer. Sci Rep 7:44301 28513626 10.1038/srep44301 PMC5434791

[38] Ahmed R et al (2023) A synthetic model simulator for intracranial aneurysm clipping: validation of the UpSurgeOn AneurysmBox. Front Surg 10:1185516 37325417 10.3389/fsurg.2023.1185516 PMC10264641

